# Enhancing Recycling Participation: Behavior Factors Influencing Residents’ Adoption of Recycling Vending Machines

**DOI:** 10.3390/bs14111071

**Published:** 2024-11-08

**Authors:** Xinyuan Zhang, Guangya Deng, Emmanuel Nketiah, Victor Shi

**Affiliations:** 1 School of Taxation, Jilin University of Finance and Economics, Changchun 130117, China; 2 College of Economics and Management, Shenyang University of Chemical Technology, Shenyang 110142, China; mingyuedeng@163.com; 3 School of Economics and Management, Nanjing University of Science & Technology, Nanjing 210094, China; emmanuel2020@njust.edu.cn; 4 Lazaridis School of Business and Economics, Wilfrid Laurier University, Waterloo, ON N2L 3C5, Canada

**Keywords:** recycling vending machines, technology acceptance model, social influence, perceived risk

## Abstract

Recycling is a crucial waste management option because of the increasing amount of waste generated and the limited space in landfills. However, traditional recycling processes, which require individuals to deliver large quantities of waste to recycling centers, can discourage participation. To address this issue, this study expanded upon the technology acceptance model (TAM) by incorporating perceived risk and social influence to examine residents’ intentions to adopt recycling vending machines. This study used partial least squares structural equation modeling based on the data collected from 525 individuals in Jiangsu Province, China. This study’s findings indicate that TAM components, such as attitudes, perceived usefulness, and perceived ease of use, positively influence residents’ intentions and behaviors to adopt recycling vending machines. Additionally, perceived usefulness and ease of use significantly affected attitudes toward recycling vending machines. This study also found that social influence had a significant positive impact on perceived usefulness and ease of use, while perceived risk negatively influenced these factors. Furthermore, attitude played a crucial mediating role, with additional factors impacting intentions and behaviors through attitude. Overall, this research can help stakeholders such as waste management companies to understand residents’ concerns and improve the implementation of recycling vending machines.

## 1. Introduction

Waste treatment is an enduring challenge for both developed and developing nations [1]. In 2016, the World Bank estimated that the global waste generation volume was 2.01 billion tons, and it is anticipated to reach 3.40 billion tons by 2050 [2]. The considerable and persistent increase in solid waste production over the past few decades indicates the depletion of natural resources. This is partly attributable to the expanding global population and per capita gross domestic product [3]. Ineffective waste management has an enormous impact on the generation of greenhouse gases, safety for the environment, and general health [4].

Waste management is integral to sustainable development, as emphasized in the 2030 Agenda for Sustainable Development adopted in 2015 [5]. Effective strategies prioritize waste avoidance or minimization and source separation of municipal solid waste, including plastic, glass, metal, and cardboard [6]. Current practices, such as using colored bags, distinct collection containers, and deposit applications, aim to segregate recyclable garbage at the source [7,8]. However, these methods still face challenges, including improper disposal by residents.

A recycling vending machine (RVM) (see Appendix B for the list of abbreviations) is a mechanized apparatus employed to gather packaging waste in a segregated manner autonomously [9]. These mechanisms can effectively mitigate human influence in the process of source separation. The increasing prominence of information technology has led to a notable focus on utilizing this technology for waste management. RVMs are commonly employed to promote responsible waste management within communities, specifically focusing on the adequate disposal of recyclable materials, including paper (cardboard cups), plastic bottles, and aluminum [10,11]. The rapid growth of urban areas in Jiangsu Province has led to an increase in the generation of municipal solid waste (MSW), putting pressure on waste management systems and infrastructure [12]. According to the National Bureau of Statistics of China (NBSC) [13], there has been a notable upward trend in municipal solid waste generation within Jiangsu Province, with the quantity rising from 7.44 million metric tons per year in 2004 to 18.71 million metric tons per year in 2020. According to the NBSC [13], the cumulative waste generation in the province between 2004 and 2020 was predicted to be 204.7 million metric tons. Jiangsu is a major industrial hub in China, leading to the generation of large quantities of industrial waste that require proper management and disposal [14]. While Jiangsu has made progress in waste recycling, there are still challenges in developing an efficient recycling infrastructure and increasing recycling rates [15]. Poor waste management practices can lead to environmental pollution, including groundwater contamination, air pollution, and the release of greenhouse gases from decomposing waste [16,17].

RVMs have significantly shifted waste management by offering accessible, interactive, and environmentally friendly options to communities. They encourage appropriate recycling behaviors, divert waste from landfills, and promote a circular economy, making them essential tools in the fight against environmental degradation. However, in rural or low-income areas, access to recycling centers may be limited. For example, under California’s “bottle bill” recycling program, while stores charge extra for beverages in containers, some residents in rural or low-income areas struggle to find convenient recycling centers [18]. Some consumers perceive recycling as time-consuming, which can act as a barrier. This is particularly true for those with higher levels of education and social status, who might have different priorities or more resources to manage their time effectively [19]. The economic benefits derived from recycling, such as receiving cash back through deposit refund programs, may not be sufficient to encourage widespread participation. Consumers might require more substantial incentives or rewards to motivate them to use RVMs regularly [20]. Therefore, the adoption of RVMs is significantly influenced by residents’ awareness and willingness to participate in such programs. A study conducted in North Bengaluru, India, highlighted that willingness to adopt RVMs was influenced by factors such as involvement, convenience, awareness, and incentives [21]. The existing literature has investigated RVM [7,9,10] and recycling intentions [22,23]. This suggests that a lack of awareness about the benefits and functionality of RVMs could hinder their adoption. Several studies have assessed the impact of perceived risk on intention [24,25,26,27] and social influence on intention [25,28,29]. However, none of the studies addressed residents’ intention to adopt RVMs. To address this research gap, this study extended the technology acceptance model (TAM) to incorporate factors such as social influence and perceived risk to better understand the factors influencing residents’ intention to adopt recycling vending machines. The rationale behind social influence and perceived risk influencing residents’ adoption of RVMs is rooted in the understanding that social norms and the influence of social networks encourage recycling behaviors [30,31,32], while perceived risks associated with recycling practices or technologies deter adoption [33,34]. These factors interact within the broader context of recycling behaviors, highlighting the need for strategies addressing social and psychological barriers to encourage greater participation in recycling initiatives.

Therefore, this present study utilizes the TAM as the primary theoretical framework. It integrates two additional factors (perceived risk and social influence) as precursors to examining the desire of residents to adopt RVMs. The TAM is a popular structure for evaluating the psychological aspects that contribute to the adoption of emerging technologies and green behaviors [35,36,37]. This model is widely accepted and valued owing to its suitability, logical consistency, and strong foundation. Considering social influence and perceived risk in extending the TAM to assess residents’ intention to adopt recycling vendor machines in China is crucial due to the country’s collectivist culture, where community opinions significantly sway individual behavior. Additionally, perceived risks, such as concerns over the reliability and environmental impact of new technologies, can be a substantial barrier to adoption. By incorporating these factors, the extended TAM can better predict adoption intentions and inform strategies to promote recycling initiatives in China.

Several studies have assessed the impact of perceived risk on intention [24,25,26,27] and social influence on intention [25,28,29]. However, none of the studies addressed residents’ intention to adopt RVMs. This study offers several significant findings that advance our understanding of the motivations driving individuals in Jiangsu Province to adopt RVMs. Notably, this research identifies critical factors that enhance policy effectiveness surrounding RVM adoption. Moreover, this study’s innovative application of the TAM provides valuable insights into the impacts of these factors on residents’ intentions to adopt RVMs, specifically by incorporating dimensions of perceived risk and social impact. The analysis reveals that attitudes play a mediating role in shaping these adoption intentions, offering a more nuanced view for policy and decision-makers. These findings contribute to developing theoretical and practical recommendations that can guide policymakers in designing more effective strategies for encouraging RVM adoption. Furthermore, they offer insights supporting eco-friendly initiatives, aligning with China’s 13th Five-Year Plan goals, prioritizing waste management improvements and reducing pollution [38]. This research also underscores the potential for RVM implementation to lower waste generation, boost recycling rates, and foster recycling industry growth through advanced waste sorting and recycling systems. Ultimately, this study serves as a valuable framework for other provinces in China, deepening our comprehension of factors influencing citizens’ readiness to embrace recycling technologies.

## 2. Literature Review and Hypotheses Development

### 2.1. Technology Acceptance Model (TAM)

The initial proposition of the TAM was put up by Davis [39], who examined users’ adoption of information technologies and drew upon the theoretical framework of the theory of reasoned action. The TAM is a widely recognized theoretical framework employed to comprehend individuals’ propensity to utilize and embrace particular information systems [40,41]. According to Williams [42], the model in question has gained significant influence and is widely regarded as a strong and efficient model within user acceptance behavior theory. The TAM is a commonly used framework for evaluating and forecasting the acceptance of technology among potential users [37,43,44]. Concurring with the TAM concept, perceived usefulness and ease-of-use perception can be used to infer intentions and behaviors when adopting an emerging green technology [45,46]. According to this theoretical framework, the decision to embrace a specific technology is contingent on an individual’s evaluation of its functional and practical advantages [36]. Current research has utilized the TAM to explore the public acceptance of automotive technologies. Huang et al. [47] inspected the influence of user knowledge on their readiness to adopt electric vehicles. Zhang et al. [48] examined consumer intention to purchase a new energy vehicle. Yuen et al. [49] explored the elements affecting the public adoption intention of autonomous vehicles. Wang et al. [44] studied the public adoption of EV charging schedules. These studies demonstrate the application of the TAM in understanding the dynamics of consumer behavior and acceptance of various automotive technologies. The adoption of diverse technologies has been widely investigated using the TAM theory in multiple studies [41,50,51]. Adu-Gyamfi et al. [36] employed the TAM as the primary theoretical framework to examine the acceptance of battery-swap technology in China. Similarly, Rejali et al. [52] used the TAM to explore the pre-existing inclination toward embracing fully autonomous vehicles within the context of Iran.

The TAM posits that an individual’s conduct can be anticipated mainly by behavioral intentions [40]. This intention is influenced by their attitudes towards the activity and the perceived social pressures to engage in it. According to [39], under the TAM, the critical determinants of residents’ desire to adopt new technologies are perceived usefulness (PU) and ease of use (PEU). According to Davis [39], PU describes the extent to which people believe using a particular technology can improve performance. PEU pertains to the degree to which individuals perceive the utilization of a particular technology to be devoid of exertion [40]. The perception of new technology being user-friendly leads to the belief that it is valuable and fosters a favorable attitude toward its use. Consequently, users’ intentions to accept new technologies are positively influenced by both PU and attitudes towards use [44,52,53].

Moreover, the public’s acceptance of recycling vending machines (RVMs) may be contingent upon factors such as the level of ease in operating them and the perceived value they bring, including economic, social, and environmental benefits. Furthermore, the efficacy of RVMs to offer convenient and expeditious means for individuals to discard their recyclable items, particularly in situations where conventional recycling bins or facilities are not readily available, while simultaneously mitigating the accumulation of waste in landfills, preserving finite resources, and curtailing pollution linked to the manufacturing of fresh materials, remains uncertain. Ultimately, the proposed initiative aims to provide rewards or incentives to cultivate an enjoyable and transformed atmosphere, fostering motivation among individuals, particularly children and teenagers, to actively engage in recycling endeavors and cultivate positive environmental behaviors from a young age.

The residents’ desire to adopt RVMs complies with the connections between expectations proposed in the TAM. Hence, this study investigates the underlying mechanisms contributing to residents’ acceptance and adoption of RVMs by utilizing the TAM as a hypothetical model. The fundamental internal links within the TAM were empirically validated through a multitude of research investigations. Therefore, we propose the following hypotheses:

**H1.**  *PU has a positive impact on residents’ intention to adopt recycling vending machines*.

**H2.**  *PU has a positive impact on attitude regarding recycling vending machines*.

**H3.**  *Attitude has a positive impact on residents’ adoption intention of recycling vending machines*.

**H4.**  *PEU has a positive impact on residents’ adoption intention of recycling vending machines*.

**H5.**  *PEU has a positive impact on attitudes regarding recycling vending machines*.

**H6.**  *PEU had a positive effect on PU*.

**Figure 1:**
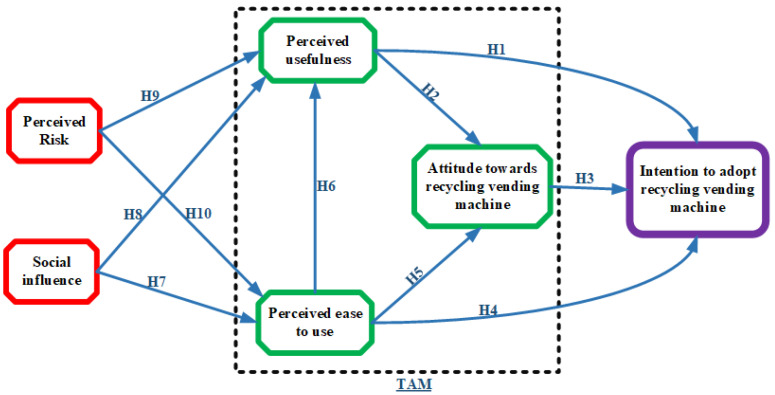
The conceptual underpinning of this research. Note: TAM = technological acceptance model.

The primary emphasis of the original TAM centers on the factors that influence the adoption of technology, namely PEU and PU, without explaining the formation of these two perceptions. Nevertheless, the TAM establishes a fundamental association that suggests the impact of external influences on user adoption by altering their cognitive beliefs, namely, PEU and PU. Williams [42] posited that the identification of antecedents influencing PU and PEU could provide a more comprehensive comprehension of the consumer adoption of technology. Numerous studies have scrutinized the link between user acceptance and external characteristics such as PU and PEU. These studies have established that external factors have a substantial effect on user acceptance [36,41,48]. The application of these findings to practical contexts can significantly enhance our comprehension of this phenomenon and furnish managers with actionable recommendations. Drawing upon a complete review of relevant studies and employing a rigorous factor scrutiny technique, this study successfully incorporated two external variables, social impact and perceived risk, into the original TAM. This integration resulted in the development of an RVM adoption model. The theoretical foundations underlying the incorporation of additional variables and establishment of the hypotheses are illustrated in Figure 1.

### 2.2. An Extended Variable of Social Influence and Perceived Risk

#### 2.2.1. Social Influence (SI)

The social influence (SI) concept pertains to the extent to which a particular user perceives that influential others hold the belief that they ought to utilize technological devices [54]. This concept is applicable to the image component of the innovation diffusion theory as proposed by Rogers [55], the subjective norm component of the theory of planned behavior formulated by Ajzen [56], the theory of reasoned action (TRA) established by Fishbein and Ajzen [57], and the social factor component of the model of personal computer utilization, as described by [58]. Prior studies have indicated that an individual’s inclination to adopt a specific technology can be impacted by external factors, as demonstrated by Wokke and Rodenrijs [59] and Abbas et al. [60]. Prior research has shown that SI benefits individuals’ perceptions of PEU and PU [44,61,62]. Nguyen [28] found that SI is positively associated with adoption intention. According to Bedard and Tolmie [63], empirical evidence proposes that SI has a favorable impact on the adoption of eco-friendly consumer behaviors. According to Bruno et al. [25], a notable positive correlation exists between SI and the intention to recycle. Schepers and Wetzels [64] discovered that a significant majority of research (96%) demonstrated a correlation between SI and PU. Additionally, 67% of the studies suggested a connection between SI and PEU. Consequently, drawing on the arguments above, the subsequent hypotheses are proposed:

**H7.**  *SI has a positive impact on PU*.

**H8.**  *SI has a positive impact on PEU*.

#### 2.2.2. Perceived Risk (PER)

PER refers to an individual’s anticipation of potential adverse results related to technology adoption [65]. Rogers [66] posited that individuals’ desire to use technology is shaped by ambiguity stemming from a lack of knowledge and the inability to forecast outcomes accurately. Likewise, scholars have recognized risk perception as a notable impediment to the acceptance and implementation of novel technologies and innovations [67,68]. The significance of this concept depends on the fact that the use of technology has inherent risks and yields uncertain and unpredictable outcomes. According to Jacoby and Kaplan [69], there are six distinct categories of perceived risk: performance, financial, social, psychological, physical, and time loss. In the context of investment platforms, the pertinent categories of risk commonly considered include performance and financial risk. Financial risk pertains to the potential for monetary losses. Performance risk is the prospect that a technological system will fail to operate effectively [40,69]. Users’ acceptance of technology may be adversely affected by a heightened perception of risk, as it can amplify worries and vulnerabilities associated with the technology [70]. According to [68], the recognition of PER plays an essential role in the acceptance of innovations and in shaping customer evaluations of the value of these innovations. Chen and Aklikokou [71] studied e-government acceptance and revealed that the PER associated with technology significantly influences PEU and PU. The present research acknowledges that risk factors encompass concerns regarding the performance and quality of RVM initiatives, the level of enthusiasm residents exhibit in utilizing them, and people’s judgments regarding the utility and usefulness of RVMs. Therefore, this concept has frequently been integrated into the TAM to enhance its scope. Previous studies have acknowledged PER as a precursor to behavioral intention [48,72], PU, and PEU [44] in previous studies. Further investigation is necessary to comprehensively understand the impact of perceived risk on the TAM, as shown by varying outcomes in prior studies [40,43]. Zhang et al. [48] revealed a significant negative influence of PER on attitude and purchase intention. According to Zhao and Khaliq [26], there is a negative link between PER and intention to use. Nazir et al. [73] and Wang et al. [74] demonstrated an adverse link between PER and behavioral intention. Katebi et al. [75] revealed that PER negatively influences usefulness and ease of use. Based on the information provided, the following hypotheses are suggested:

**H9.**  *PER has a significant effect on the PU*.

**H10.**  *PER has a significant effect on the PEU*.

### 2.3. Attitude as a Mediator

The intermediary element of attitude has the potential to impact residents’ intentions to use technology effectively. The outcome presented here is a consequence of the manner in which individuals interpret the appeal of convincing messages [76,77,78,79,80,81,82]. Domínguez-Valerio et al. [83] discovered no direct correlation between sustainable knowledge and sustainable behavior. However, the outcomes indicate that attitude plays a vital part in mediating the connection among these two factors. Bananuka et al. [84] revealed that attitude mediated the influence of users’ subjective norms on adoption behavior. Adu-Gyamfi et al. [36] determined that attitude has a mediating role in the link between knowledge and the intention to adopt battery-swap technology. Chen et al. [41] discovered that attitude impacts the link between PEU, PU, and behavioral intention. Mustafa et al. [53] revealed that attitude mediates the connection between PEU, PU, and intention. Based on the previously discussed results and theoretical frameworks, the following research hypotheses are articulated:

**H11.**  *PU via a positive attitude can impact residents’ intention to adopt recycled vending machines*.

**H12.**  *PEU via a positive attitude can impact residents’ intention to adopt recycled vending machines*.

### 2.4. Perceived Usefulness (PU) and Perceived Ease of Use (PEU) as a Mediator

As the TAM emphasizes, perceived usefulness and perceived ease of use are critical elements influencing user acceptance of information technology [39]. Numerous domains, including online marketplaces [85], mobile applications [86], and e-commerce [87], have extensively studied these constructs, consistently demonstrating their significant impact on customer intention to use technology. Compared with perceived ease of use, perceived usefulness shows a stronger correlation with usage behavior, suggesting that it plays a crucial role in determining user acceptance. The TRA component of social influence affects user acceptance by forming attitudes and arbitrary norms [88]. Further research is needed to understand how the social impact directly influences the perceived benefits and ease of use of recycled vending machines. The precise mechanism by which social impact mediates the relationship between perceived usefulness, perceived ease of use, and intention to use recycled vending machines remains unclear despite statistical evidence suggesting that attitudes and subjective norms may influence intentions. Research demonstrates that perceived risk, a crucial determinant of technological acceptability, adversely influences purchase intentions [33]. Perceived risk in the context of recycled vending machines can include concerns about the reliability of recycled goods, the environmental impact of recycling practices, and the reliability of vendors. Research suggests that perceived risk may mediate the interaction between purchase intention and personal norms, mediating the relationship between residents’ intentions to adopt recycled vending machines and social influence.

The integration of trust and risk into the TAM framework has been proposed to better predict consumer acceptance of e-commerce [89]. This approach could be adapted to elucidate residents’ intentions to adopt recycled vending machines by considering trust in the vendor and the perceived risks associated with recycling. Trust could potentially mediate the relationship between PU, PEU, and intention to adopt recycled vending machines, as trust in the vendor’s commitment to sustainability might enhance perceived usefulness and ease of use. Perceived usefulness and perceived ease of use are critical mediators between social influence, perceived risk, and residents’ intentions to adopt recycled vending machines. Perceived usefulness directly influences the intention to use technology [39], whereas perceived ease of use may indirectly influence this intention through PU [90,91]. Further research is necessary because of the direct impact of social influence on perceived usefulness and ease of use. Social influence influences attitudes and subjective norms, impacting intentions [88]. Perceived risk negatively impacts purchasing intention [33], implying that it could serve as a mediator between intention and social influence. By reducing perceived risk and increasing trust in sellers, trust may mediate the relationship between perceived usefulness, ease of use, and intention, thereby promoting the acceptability of recycled vending machines. Based on the information provided, the following hypotheses are proposed:

**H13.**  *Perceived usefulness mediates the relationship between social influence and intention to recycle vending machines*.

**H14.**  *Perceived ease of use mediates the relationship between social influence and intention to adopt recycled vending machines*.

**H15.**  *Perceived usefulness mediates the relationship between perceived risk and the intention to adopt recycled vending machines*.

**H16.**  *Perceived ease of use mediates the relationship between perceived risk and the intention to adopt recycled vending machines*.

## 3. Materials and Methods

### 3.1. Study Area

Municipal waste generation has risen yearly in Jiangsu Province over the past decade [92]. In 2019, Jiangsu Province exhibited a substantial waste generation rate, surpassing that of prominent urban centers such as Beijing and Shanghai. The province’s waste output amounted to 18.1 million tons, positioning it as the second-highest contributor to waste generation [93]. A strategy to build zero-waste communities to reduce solid waste, promote waste recycling, and minimize landfill waste was recently announced by the Department of Ecology and Environment of Jiangsu Province [94]. Hence, recycling vending machines catalyze the promotion of recycling behavior among individuals because they offer a practical and readily available means of disposing of recyclable items. This practice can potentially mitigate the quantity of waste deposited in landfills, save finite resources, and mitigate pollution connected with the manufacture of fresh materials. Also, the implementation of recycling vending machines has proven to be particularly effective in fostering a culture of recycling among the younger generations. A stimulating and transformed environment is established by implementing rewards or incentives, fostering motivation among individuals, particularly children and teenagers, to engage in recycling initiatives and cultivate positive environmental behaviors from a young age. Not only do they provide individuals with the opportunity to dispose of their recyclable materials responsibly, but they also engender a favorable environmental impact and contribute to the circular economy. Therefore, policymakers should pay particular attention to recycling vending machines for waste management because they pertain to the achievements of the circular economy and solutions to the waste problem. Over the past decade, significant developments have occurred in the recycling infrastructure and educational campaigns in Jiangsu Province [94]. However, effectively reducing the quantity of trash disposed of in landfills remains a significant challenge. Therefore, this study utilizes the TAM as the primary theoretical framework and includes two additional constructs (social influence and perceived risk) that impact residents’ intention to adopt recycling vending machines. Social influence significantly impacts recycling behavior, as people often follow peer and community norms when making environmental decisions. Peer influence plays a crucial role in decisions about using recycling vending machines, which can encourage others through their presence in public spaces [25]. Additionally, environmental concerns can indirectly boost the intention to use smart recycling systems by increasing their perceived usefulness [95]. Perceived risks, such as concerns over product quality, contamination, or time required to use the machines, can deter or encourage adoption [24]. When risks are low and machines are socially accepted, adoption rates increase. Addressing these risks through education can further improve uptake. This research aimed to ascertain the relative efficacy of primary components based on public opinion. Additionally, we sought to establish the degree to which the present state of these characteristics genuinely impacted citizens’ inclination to embrace recycling vending machines. This study analyzed the elements influencing residents’ intention to adopt recycling vending machines, considering the underlying assumptions and reflecting real-life situations.

### 3.2. Survey Participants and Data Collection Process

The survey was administered online using the Questionnaire Star platform (www.sojump.com) from mid-June to mid-August 2023. This platform boasts a substantial user base of over one million active individuals and operates across 345 cities in China, ensuring a broad geographic reach. Questionnaire Star has established a strong reputation for reliable survey distribution and collection, employing an integrity scoring system to screen respondents effectively and ensure their suitability for participation. The platform’s accessibility via smartphones further enhances its reach, allowing for a diverse representation of respondents in terms of income, education, and age.

The preliminary survey was initially prepared in English and subsequently translated into Chinese by a professional linguist, as the questionnaire items were sourced from the English-language literature without modifications. A five-point Likert scale was employed, with responses ranging from 1 (strongly disagree) to 5 (strongly agree). During the pre-test stage, two items were excluded due to incorrect wording and excessive length [96]. The survey was distributed to the target audience through random sampling to enhance generalizability and reduce bias [97,98,99]. The URL was shared via various media channels, encouraging participants to forward it for broader participation.

This explanatory study initially involved 550 individuals, of which 530 responses were utilized after excluding five unsuitable entries that did not meet the survey criteria. The elimination process ensured that only complete and relevant responses were included, enhancing the quality and reliability of the analyzed data and meeting the recommended guideline of at least 10 cases per unit [100].

### 3.3. Measurement Instrument

The items pertaining to each component of the measure were sourced from previous studies with some mild adaptations to align them with the specific focus of this study. The constructs of attitude, PEU, and PU, as outlined by Davis [39], were utilized in this study. The constructs were obtained from independent sources, Adu-Gyamfi et al. [36] and Zhang et al. [48]. The concept of perceived risk is derived from Katebi et al. [75] and Joo et al. [101]. Social influence was assessed using the scale established by Su et al. [61]. The selection of elements pertaining to the inhabitants’ intention to adopt recycling vending machines adhered to the guidelines proposed by Ajzen [102] and Wang et al. [103]. All constructs employed in this study were measured by at least three items on a five-point Likert scale, as documented in Appendix A.

### 3.4. Data Analysis

This study employed partial least squares structural equation modeling (PLS-SEM) to assess the validity of the theoretical framework. SmartPLS4, a statistical method, was utilized for this determination [79,104]. Statistical tests conducted using the SPSS software 28 were found to have a level of significance of 0.05. The Kaiser–Meyer–Olkin (KMO) test was employed to assess the appropriateness of the data for element assessment. The obtained KMO value of 0.894 surpassed the recommended threshold of 0.60 [105], indicating that the data were deemed suitable for this study. Furthermore, the statistical analysis revealed that the outcomes of Bartlett’s sphericity test exhibited a high level of significance, with a *p*-value of less than 0.001. PLS-SEM can be compared to multivariate regression, as it aims to optimize the explanatory variance of the endogenous variable [106,107,108]. The assessment involves the integration of the main factor regression with ordinary least squares regression, as demonstrated in previous studies [109,110]. PLS-SEM is often regarded as the most appropriate model for elucidating intricate phenomena. Therefore, this research employs the SEM procedure, wherein each variable provided in this investigation is assessed using numerous statements, as recommended by Hair et al. [106]. The utilization of the partial least squares structural equation modeling approach, incorporating the TAM and its antecedent factors, is deemed suitable for predicting residents’ intention to adopt recycling vending machines. PLS-SEM is a variance-based technique that is deemed more suitable for testing explanatory models, in contrast to standard covariance-based structural equation modeling, which primarily focuses on verifying the current theory [104]. This research introduces social influence and perceived risk as novel factors that have not been previously validated in the context of residents’ intentions to embrace recycling vending machines. This study used PLS-SEM to examine the relationships between these variables.

### 3.5. Descriptive Statistics

Of the 525 samples, 238 were male and 287 were female. Of the total sample size, 429 participants had a Bachelor’s degree or higher, suggesting that a significant proportion of the respondents had attained a comparatively advanced education level. It is noteworthy that a significant proportion of the respondents (83.4%) were located in urban areas. A significant proportion of the people, including more than half, consisted of respondents aged between 26 and 45 years, with 44.8% falling within the 26–35 age range and 32.4% falling within the 36–45 age interval. Approximately 42.3% of the participants in the survey described themselves as private-sector employees, while 22.9% reported being employed in the public sector. These figures provide insights into the prevailing employment patterns in China. The participants’ income levels varied from CNY 5000 to CNY 20,000 per month (about USD 688 to USD 2750), with 235 individuals falling between CNY 5000 and CNY 10,000 per month (approximately USD 688 to USD 1372.3). Table 1 exhibits an extensive breakdown of the demographic statistics with greater specificity.

## 4. Results

Chin [111] proposed a methodology that advocates a two-stage approach to the interpretation of data obtained by PLS-SEM. It is imperative to evaluate the measurement model by executing the prescribed PLS procedure per 300 iterations. The estimation of the structural analysis model and level of confidence determination of route scrutiny was conducted using bootstrapping per 5000 subsamples, as recommended by Hair et al. [112] and Anderson and Gerbing [113].

### 4.1. Model for Measurement

**Figure 2:**
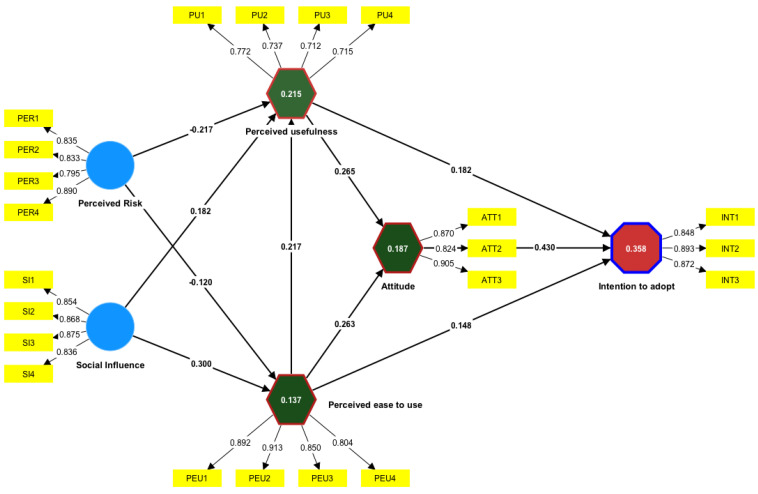
Outcome of the study model (hypothesis).

The measurement model was assessed following the guidelines provided by Hair et al. [110]. The item discriminant validity and reliability were evaluated to assess the overall adequacy of the measurement technique. The model consists of a total of 22 observable variables (OVs) that collectively create six latent variables (LVs). Specific model pathways are shown in Figure 2.

Internal consistency was assessed using composite reliability and Cronbach’s alpha. Cronbach’s alpha and composite reliability (CR) were used because of their capacity to generate more accurate reliability outcomes by incorporating elements with weights determined by the construct factor loadings [110]. The composite reliability (CR) scores ranged from 0.714 to 0.895. This study revealed that Cronbach’s alpha (α) values ranged from 0.715 to 0.888, as indicated in Table 2. These findings indicate the internal consistency of the measures above the threshold of 0.7 [114,115].

The assessment of the measurement strategy convergent validity involved an examination of the factor loadings and AVE. Hair et al. [110] suggest that it is desirable for indicator loadings to exceed 0.70. The construct is responsible for over 50% of the variability in the item, as indicated by this finding. The indicator loadings in this investigation were all above 0.70, as shown in Table 2. Moreover, the average variance extracted (AVE) of the construct should surpass the threshold of 0.5, as suggested by [116]. According to the findings presented in Table 2, the AVE values for each construct range from 0.539 to 0.759. These values were consistent with the commonly accepted rule of thumb.

An examination of discriminant validity is necessary to establish that a construct is distinct from others [117]. The Fornell and Larcker criterion and the heterotrait–monotrait (HTMT) ratio were utilized to assess discriminant validity in Table 3. According to Fornell and Larcker [116], discriminant validity is deemed adequate when the square root of the average variance extracted (AVE) exceeds the correlation values between constructs. Numerous studies have suggested that the Fornell and Larcker criterion may exhibit diminished efficacy when employed to ascertain the discriminant validity. Consequently, Ab Hamid et al. [118] opted to utilize the HTMT of the correlation criterion to assess the reliability and coherence of the findings. Henseler et al. [119] posited that the optimum HTMT value should be less than 0.85, which aligns with the outcomes of our measuring methodology (refer to Table 3). Multicollinearity was assessed to mitigate bias in the regression results. The variance inflation factor (VIF) values provided empirical support, indicating that the structural model’s outcomes were unaffected by collinearity. Rendering to Hair et al. [110], the VIF values were below 3.0. No collinearity was observed in this study, as all VIF values were below the cutoff point of 3.0, as indicated in Table 2.

### 4.2. Structural Model

Once the measurement model has been constructed and validated, the subsequent step involves analyzing the structural model. In contrast to CB-SEM, PLS-SEM places greater emphasis on the explanatory capacity of the model. Consequently, the coefficient of determination (R^2^), which quantifies the proportion of variance in the endogenous construct explained by exogenous variables, is regarded as a significant indicator of a model’s predictive accuracy. According to Cohen’s recommendations [120], R^2^ values of 0.02, 0.13, and 0.26 can be classified as small, medium, and large, respectively. Hair et al. [121] observed significant variations in the R^2^ measures across diverse study areas. In consumer behavior studies, R^2^ values exceeding 0.2 are deemed to indicate strong levels of explanatory power. The R^2^ value of the conceptual framework of INT in this study was 0.358, while the R^2^ value of the original TAM of INT was 0.355. These values are considered satisfactory compared to other published research on behavioral intention, where R^2^ ranged from 0.190 to 0.241 using PLS-SEM modeling [122,123]. Hair et al. [110] utilized a cross-validated redundancy metric Q2 to gauge the predictive capacity of the structural model. The Q2 value must be greater than zero. The blindfolding technique was implemented in this study with an omission distance of 6. The Q^2^ value for the conceptual framework of INT was 0.423, while the Q^2^ value for the original TAM (PEU, ATT, and PU) of INT was 0.415. The findings demonstrate the predictive accuracy of the models, which was evaluated using the SRMR proposed by Hair et al. [104]. The SRMR values for the conceptual framework and the original TAM were 0.060 and 0.066, respectively. These values were below the threshold of 0.08, indicating that the models met the requirements for fit, as shown in Table 4.

The significance level and magnitude of path coefficients were subsequently evaluated using the bootstrapping method. The standard deviations were estimated using a bootstrap method, whereby 5000 sub-samples were generated by randomly selecting observations from the original dataset. The results from the study of direct structural relationships indicate that all hypothesized relationships exhibit statistical significance at the 1% level (*p* < 0.01) in relation to residents’ intention to adopt recycling vending machines (INT), with the exception of H10, which was significant at the 5% level. We found that ATT, PEU, and PU significantly positively affect residents’ intention to adopt recycling vending machines. We also discovered that PER significantly adversely impacts PU and PEU. We also found that SI positively and substantially impacts recycling vending machines’ PU and PEU. On the other hand, PU and PEU were positively correlated with attitudes toward recycling vending machines. This study found that PEU positively significantly affects the perceived usefulness of recycling vending machines. Therefore, all the hypotheses were analytically carried out (see Table 5).

### 4.3. Mediation Analysis

Subsequently, the scrutiny appraised a comprehensive model incorporating the mediator and examined the statistical significance of the indirect effect. This study observed that the influence of antecedent factors on residents’ desire to use recycled vending machines was mediated by attitude, PU, and PEU. These indirect effects are statistically significant at the 5% level, as shown in Table 6. Hence, this study successfully identified the presence of partial mediation effects among each construct and residents’ intention to adopt recycling vending machines. This study found that social influence and perceived risk significantly impacted residents’ intention to adopt recycling vending machines through perceived usefulness (PU) and perceived ease of use (PEU). Social influence and encouragement from peers or the community positively impacted residents’ PU of the machines, making them view the technology as more beneficial. On the other hand, perceived risks, such as concerns about the reliability or safety of the machines, negatively affected the PEU. These factors shaped residents’ attitudes toward adopting recycling vending machines, emphasizing the need to minimize perceived risks and leveraging social support to enhance adoption. The categorization of mediation effects into complementary and competing mediation can be determined based on the paths of indirect and direct effects, as discussed by Hair et al. [110] and Zhao et al. [124]. In cases where the direct effect exhibits a sign contrary to that of the indirect impact, competitive mediation is observed. This phenomenon suggests that the middle factor diminishes the strength of the association concerning independent and dependent factors. The findings in Table 6 indicate that attitude serves as a complementary mediator in the link between PU, PEU, and residents’ intention to adopt recycling vending machines.

## 5. Discussion

This study contributes to the literature on technology adoption and waste management by testing an extended technology acceptance model (TAM) framework regarding residents’ intention to adopt recycling vending machines. Although the TAM has been tested in several other contexts of technology adoption, to our knowledge, the current research represents the first attempt at modeling psychological factors that lead to residents’ intention to adopt recycling vending machines. Several studies have considered psychosocial factors related to the adoption of technology, and, when a comparison is made, the findings seem to align.

This study supports the original TAM framework established by Davis [39]. The three antecedents of intentions–attitudes, PU, and PEU explain behavioral intentions. Perceived usefulness directly affects residents’ intention to adopt recycling vending machines. This is consistent with Zhang et al. [48], Cudjoe et al. [125], and Su et al. [61], who showed that PU positively impacts behavioral intentions. The study further found that perceived usefulness influences attitude towards recycling vending machines. This implies that residents may consider RVMs helpful because they offer a practical and accessible alternative to recycling various items. Residents have a stronger intention to adopt recycling vending machines because they feel they are useful, are more likely to acquire good attitudes regarding their use, and are more likely to do so. They might see the devices as a sensible and efficient response to their recycling requirements, which might inspire them to make the necessary preparations to embrace and use the technology.

Attitude displays the strongest direct impact on residents’ intention to adopt recycling vending machines. The outlines found in the present study are consistent with other findings, such as Wang et al. [44], Mustafa et al. [53], and Lin and Guan [117]. This indicates that residents have a positive attitude toward recycling vending machines and are more likely to view them as practical, convenient, and environmentally friendly.

PEU directly affects residents’ intention to adopt recycling vending machines. This is how easily used individuals perceive a system or technology to be, and it directly and positively affects adoption intentions. PEU positively affects attitudes and PU. Residents’ opinions toward utilizing recycling vending machines improve when they perceive them as easy to use. The idea that simplicity of use lowers the work and complexity of the recycling process, making it more accessible to a larger audience, is the primary motivator behind this positive mentality. Furthermore, PEU increases the machines’ perceived usefulness (PU), supporting the notion that operating simplicity raises opinions about their efficacy and usability. This confirms the results of Liu and Hsu [95], who found that PEU positively influences intentions, whereas Wang et al. [126] found a positive impact of PEU on attitude and PU. These findings highlight a reinforcing cycle: ease of use improves attitude and perceived usefulness, which in turn boosts the intention to adopt RVMs. Finally, residents find recycling vending machines appealing not only because they are easy to use but also because they offer a practical, effective solution for managing recyclable waste. This dual benefit ease and practicality makes them more likely to be embraced as a sustainable practice for environmental conservation.

SI was found to have a positive influence on PEU and PU. When individuals observe others using and benefiting from these machines, their perception of the machines’ ease of use and usefulness increases. This confirms the findings of Chen et al. [41], where social influence positively influences PEU, reinforcing the idea that peer usage can reduce perceived complexity. Su et al. [61] found a positive impact of social influence on PU. This implies that when individuals see others benefitting from using machines and perceive them to be useful, they can influence their perception of machines’ usefulness [127]. Perceived risk has a negative impact on the PEU and PU. This implies that when individuals perceive a high level of risk, such as concerns about the accuracy of the recycling process or potential harm to the environment owing to improper disposal, they may perceive the machines as less valuable. These results confirm the findings of Charlesworth et al. [128] and Nazir et al. [73], which also identified perceived risk as a key deterrent in adopting technology.

The findings indicate that attitude mediates PEU, PU, and residents’ intention to adopt recycling vending machines. This indicates that although adoption intentions are directly impacted by PEU and PU, the development of a positive attitude is a crucial process. PEU has a positive attitude-influencing effect because people are more inclined to think favorably of a system if they find it easy to use. In a similar vein, PU directly affects attitude; people are more likely to adopt a favorable attitude toward the system when they believe it to be helpful. These outcomes are consistent with earlier research on attitudes that moderate PU, PEU, and adoption intentions [41,53]. The mediating role of attitude emphasizes that the adoption of technology involves more than just perceived usability or functionality. Rather, it affects how those perceptions influence attitudes in general. The fact that PEU, PU, and adoption intention are positively correlated shows that encouraging vending machine recycling’s usability and ease of use is essential to boosting adoption. Locals will embrace these devices because they are easy to use or practical and because of the positive attitudes they generate, which in turn motivate usage intentions.

## 6. Conclusion and Policy Implications

### 6.1. Conclusions

With the structural and measurement model results, all path analyses that influenced residents’ intention to adopt recycling vending machines were significant. Technology acceptance model components, such as attitudes, PU, and PEU, positively impact residents’ intention to adopt recycling vending machines. Further, we found that PU and PEU significantly influenced attitudes regarding recycling vending machines. Furthermore, from an influence perspective, while social influence has a significant positive effect on perceived usefulness and PEU, perceived risk negatively influences PU and PEU. To explore the role of attitude further, we conducted a mediation analysis. We showed that attitude is a significant mediating variable and that additional factors influence intention from the perspective of attitude.

Despite acknowledging the limitations of this research, the highlighted constraints discussed in this section may serve as valuable sources of inspiration for future studies. One of the primary limitations of this study was its scope. The generalizability of our findings to diverse cultural or geographical contexts may be limited because of the specific data collection conducted in Jiangsu Province, China. Subsequent research could employ a cross-sectional approach to validate our findings. Furthermore, it is worth noting that the scope of our study is limited to the timeframe from mid-June to mid-August in 2023. As a result, future research endeavors should consider employing a longitudinal approach to investigate the factors influencing residents’ inclination to adopt recycling vending machines over an extended period. This study’s path coefficients are low, indicating potentially weak relationships between the variables. Future research should aim to enhance data collection methods to achieve higher path coefficients that meet rigorous academic standards and support more robust analytical conclusions. Furthermore, the framework employed in this research encompasses a restricted set of constructs to elucidate the factors that influence residents’ inclination to embrace recycling vending machines. Future research attempts should consider the integration of additional pertinent theories and elements to enhance the predictive accuracy of residents’ inclination to utilize recycling vending machines. Future research should examine the development of targeted policies and educational interventions aimed at increasing awareness of RVM capabilities. Such interventions could help bridge knowledge gaps, empower communities, and support effective decision-making.

### 6.2. Policy Implications

#### 6.2.1. Theoretical Implications

This study contributes significantly to the current debate on the adoption of developing technologies that support environmental sustainability by expanding the TAM. The present study reaffirmed and validated the enduring use of the TAM in examining the acceptance and intention to adopt developing technologies. In this study, we examined the psychological characteristics of individuals that contribute to the adoption of recycling vending machines. As such, this study makes a valuable contribution to the emerging field of pro-environmental behavior research by focusing specifically on the participant perspective.

This study provides practical evidence highlighting the significant influence of perceived usefulness and attitude on residents’ propensity to adopt recycling vending machines, both as direct factors and as mediators. This study examines the impact of latent factors, specifically social influence and perceived risk, within the domain of developing technologies such as recycling vending machines. It focuses on the relationship between attitudes and intentions, building on existing research in this area. Furthermore, this research extends the importance of the existing body of knowledge on beliefs, attitudes, and intentions in the field of pro-environmental studies, particularly in relation to the implementation of reverse vending machines for recycling. This study provides additional support and validation to the existing theoretical frameworks in this area. The conceptualizations and findings presented in this study contribute to and support prior research in the fields of marketing and management. This research demonstrates the mediating function of attitude and PU. While the current study could not provide conclusive evidence regarding the effect of social influence and PER on user attitudes in this particular setting, it was observed that these variables also affected the perceived usefulness of recycling vending machines. In addition, these factors had a subsequent effect on residents’ willingness to use recycling vending machines for recycling purposes. The empirical findings provide clarification on the essential roles of PU and attitude as direct and mediating factors within the framework. Furthermore, it is worth noting that there is a noteworthy impact in the literature resulting from the enclosure of other factors such as SI and perceived risk on residents’ intention to adopt novel technologies such as recycling vending machines in Jiangsu Province, China.

#### 6.2.2. Practical Implications

Perceived usefulness directly affects residents’ intention to adopt recycling vending machines. When individuals view these machines as valuable and efficient for recycling, their likelihood of adopting them increases. Policymakers and companies should implement policies to improve how communities view recycling vending machines as being valuable. Policymakers should promote the creation and installation of recycling vending machines that residents find highly beneficial. This encompasses the machines’ technical capabilities, location, user-friendliness, and seamless incorporation into everyday life. Policymakers should also consider including these design features in RVMs to enhance their appeal and efficacy. The government should also offer financial incentives for businesses to install these machines and ensure sufficient infrastructure for their operation. Additionally, policies mandating recycling or offering tax breaks to companies supporting recycling can foster the adoption of recycling vending machines. Continuous monitoring and evaluation are essential to ensure these machines are effective. Feedback mechanisms should be implemented to collect residents’ opinions on their functionality and usefulness, allowing for iterative improvements to the technology and deployment strategy.

Attitude displays the most substantial direct impact on residents’ intention to adopt recycling vending machines. Policymakers and waste management companies can design interventions and communication campaigns that specifically target residents’ attitudes toward recycling vending machines. This can be accomplished through educational initiatives and public relations efforts, and the advantages of these devices are highlighted. The likelihood that locals will adopt machines would undoubtedly increase by altering their views. Policymakers can strive to promote a recycling culture within communities. Public education campaigns, community involvement projects, and collaborations with regional groups can help achieve this goal. Residents are more likely to adopt positive attitudes toward recycling vending machines and incorporate them into their daily lives by fostering an environment that values recycling and sustainability.

PEU directly affects residents’ intention to adopt recycling vending machines. PEU positively affects attitudes and PU. This study suggests that policymakers and waste management companies ensure that recycling vending machines have logical layouts and user-friendly interfaces. They can contain unambiguous directions, sensible button placements, and understandable graphics. It is possible to boost citizens’ willingness to utilize machines by putting the user experience first and making them simple to use. This study suggests that governments should also consider how recycling vending machine placement and accessibility might be improved. It may be easier for residents to use these devices if they are situated in conveniently accessible settings such as homes or community centers. Moreover, policymakers should ensure that machines are accessible to individuals with disabilities and provide options and accommodations for a diverse population.

SI was found to have a positive influence on PEU and PU. Hence, policymakers and waste management companies must discuss adopting social influence strategies, including testimonials, positive messages, and endorsements from influential individuals or community leaders, to augment the perceived ease of use and utility of recycling vending machines. Through social influence, it becomes feasible to facilitate the widespread acceptance and implementation of these machines, thereby establishing a favorable societal norm pertaining to recycling behavior.

PER has a negative impact on the PEU and PU. Policymakers and waste management companies should deliberate measures to mitigate potential concerns and assuage perceived risks. These efforts may involve disseminating precise and comprehensive information regarding the safety, dependability, and environmental ramifications of the machines. Effectively conveying the implemented security protocols and addressing privacy concerns can bolster individuals’ trust and diminish their perceived level of danger. Proactively mitigating perceived risks makes it feasible to enhance residents’ awareness of machines’ ease of use and utility, thereby fostering their adoption.

The findings indicate that attitude mediates PEU, PU, and residents’ intention to adopt recycling vending machines. Therefore, it is imperative for policymakers and investors to actively advocate the widespread implementation of recycling vending machines to bolster citizens’ perceptions and acceptance of these machines. This can be achieved by highlighting the convenience and practicality of its use. Emphasizing the advantages of utilizing these machines makes it feasible to shape citizens’ perceptions, attitudes, and intentions, resulting in heightened adoption and efficient recycling initiatives.

## Figures and Tables

**Table 1:** Demographic statistics.

Variable	Group	Frequency	Percent
Gender	Male	238	45.3
	Female	287	54.7
Age	Under 25	55	10.5
	26–35	235	44.8
	36–45	170	32.4
	46–55	56	10.7
	Above 55	9	1.7
Employment Status	Student	98	18.7
	Self-employed	70	13.3
	Government worker	120	22.9
	Private company worker	222	42.3
	Unemployed	15	2.9
Educational Level	Junior high school and below	42	8
	High school	54	10.3
	Bachelor’s degree	215	41
	Master’s degree	189	36
	Ph.D. degree or above	25	4.8
Income	CNY 5000 or below	55	10.5
	CNY 5000–10,000	235	44.8
	CNY 10,000–15,000	170	32.4
	CNY 15,000–20,000	56	10.7
	CNY 20,000 and above	9	1.7
Location	Urban	438	83.4
	Suburban	87	16.6
	Total	525	100

**Table 2:** Reflective models: reliability measurements.

Constructs	Indicator Code	Outer Loadings	α	CR	AVE	VIF
Attitude	ATT1	0.870	0.834	0.836	0.752	2.266
	ATT2	0.824				1.623
	ATT3	0.905				2.560
Intention to adopt	INT1	0.848	0.842	0.844	0.759	1.674
	INT2	0.893				2.462
	INT3	0.872				2.319
Perceived risk	PER1	0.835	0.860	0.878	0.704	1.979
	PER2	0.833				2.024
	PER3	0.795				1.797
	PER4	0.890				2.337
Perceived ease of use	PEU1	0.892	0.888	0.895	0.749	2.246
	PEU2	0.913				2.754
	PEU3	0.850				2.035
	PEU4	0.804				1.877
Perceived usefulness	PU1	0.772	0.715	0.714	0.539	2.449
	PU2	0.737				2.371
	PU3	0.712				1.687
	PU4	0.715				1.681
Social influence	SI1	0.854	0.881	0.884	0.737	2.183
	SI2	0.868				2.869
	SI3	0.875				2.816
	SI4	0.836				2.018

**Table 3:** Discriminant validity using the Fornell–Larcker criterion and HTMT ratio.

HTMT ratio—Matrix							
No.	Constructs	1	2	3	4	5	6
1	Attitude						
2	Intention to adopt	0.647					
3	Perceived risk	0.350	0.383				
4	PEU	0.409	0.413	0.288			
5	PU	0.457	0.494	0.447	0.419		
6	Social influence	0.355	0.372	0.515	0.396	0.446	
Fornell–Larcker criterion							
No.	Constructs	1	2	3	4	5	6
1	Attitude	0.867					
2	Intention to adopt	0.546	0.871				
3	Perceived risk	−0.292	−0.326	0.839			
4	PEU	0.353	0.361	−0.255	0.866		
5	PU	0.354	0.384	−0.354	0.337	0.734	
6	Social influence	0.303	0.320	−0.448	0.354	0.357	0.859

Note: PEU = perceived ease of use; PU = perceived usefulness; and HTMT = heterotrait–monotrait ratio.

**Table 4:** Model fit.

Items	Standard Value	Present Research Model	TAM Model
SRMR	<0.08	0.060	0.066
NFI	>0.80	0.873	0.850
R^2^_Intention_		0.358	0.355
Q^2^_Intention_		0.423	0.415

Note: SRMR = standardized root mean square residual; NFI = normed fit index; TAM = technology acceptance model.

**Table 5:** Hypothesis testing results.

Hypothesis	Relationship	Estimate	Standard Deviation	*t* Statistics	*p* Values	Outcome
H1	PU → INT	0.182	0.039	4.656	0.000	Significance
H2	PU → ATT	0.265	0.039	6.838	0.000	Significance
H3	ATT → INT	0.430	0.038	11.175	0.000	Significance
H4	PEU → INT	0.148	0.037	4.057	0.000	Significance
H5	PEU → ATT	0.263	0.042	6.325	0.000	Significance
H6	PEU → PU	0.217	0.043	5.088	0.000	Significance
H7	SI → PEU	0.300	0.045	6.596	0.000	Significance
H8	SI → PU	0.182	0.046	3.952	0.000	Significance
H9	PER → PU	−0.217	0.046	4.710	0.000	Significance
H10	PER → PEU	−0.120	0.047	2.587	0.010	Significance

Note: PU = perceived usefulness; INT = intention to adopt; ATT = attitude; PEU = perceived ease of use; SI = social influence; and PER = perceived risk.

**Table 6:** Mediation model (hypothesis).

Direct Effect				Indirect Effect			*p* Values	Mediation Type	Decision
Connections	Estimate	*t* Statistics	H	Connections	Estimate	*t* Statistics			
PU → INT	0.184 ***	4.771	H11	PU → ATT → INT	0.115 ***	5.503	0.000	Complementary mediation	Supported
PEU → INT	0.153 ***	4.265	H12	PEU → ATT → INT	0.113 ***	5.543	0.000	Complementary mediation	Supported
PER → INT	−0.104 ***	4.427	H13	PER → PU → INT	−0.040	3.176	0.002	Complementary mediation	Supported
			H14	PER → PEU → INT	−0.018	2.109	0.035	Complementary mediation	Supported
SI → INT	0.152 ***	5.935	H15	SI → PU → INT	0.044	3.372	0.001	Complementary mediation	Supported
			H16	SI → PEU → INT	0.033	3.020	0.003	Complementary mediation	Supported

Note: *** *p* < 0.001.

## Data Availability

Data will be made available upon request.

## Appendix A

**Table A1:** The measurement item and source.

Constructs	Measurement Item	Source
	This part of the questionnaire employs the Likert scale. Each construct was measured with multiple items using a five-point Likert scale (1 = strongly disagree to 6 = strongly agree). 1 = Strongly disagree 2 = Very disagree 3 = Moderately disagree 4 = Moderately agree 5 = Very agree	
Attitude	ATT1—I have a positive attitude toward using recycling vending machines.	[36,39]
	ATT2—I believe using recycling vending machines is an environmentally responsible choice.	
	ATT3—I feel confident that recycling vending machines contribute to a sustainable future.	
Intention to adopt	INT1—I intend to use recycling vending machines in the near future.	[102,103]
	INT2—I am willing to make an effort to adopt recycling vending machine practices.	
	INT3—I am open to adopting recycling vending machine practices in my daily routine.	
Perceived risk	PER1—I believe using recycling vending machines poses a risk to my personal information security.	[75,101]
	PER2—I perceive a risk in terms of the reliability of recycling vending machines.	
	PER3—I think there is a risk of encountering technical issues when using recycling vending machines.	
	PER4—I am concerned about the safety of my transactions when using recycling vending machines.	
Perceived ease of use	PEU1—Using recycling vending machines is straightforward and easy.	[39,48]
	PEU2—I find it easy to understand how to operate recycling vending machines.	
	PEU3—Recycling vending machines are user-friendly in my opinion.	
	PEU4—I feel confident in my ability to use recycling vending machines effectively.	
Perceived usefulness	PU1—Using recycling vending machines makes recycling more convenient for me.	[39,128]
	PU2—I find recycling vending machines to be an efficient way to recycle.	
	PU3—Recycling vending machines make it easier for me to contribute to environmental sustainability.	
	PU4—I believe recycling vending machines are a valuable addition to recycling programs.	
Social influence	SI1—I am influenced by friends and family to use recycling vending machines.	[61,129]
	SI2—Peer recommendations have an impact on my decision to use recycling vending machines.	
	SI3—The opinions of my social circle positively affect my attitude towards recycling vending machines.	
	SI4—I am more likely to use recycling vending machines if I see others in my community doing so.	

## Appendix B. List of Abbreviations

**Abbreviation**	**Full Meaning**
ATT	Attitude
SI	Social Influence
PU	Perceived Usefulness
PEU	Perceived Ease of Use
PER	Perceived Risk
INT	Intention To Adopt
RVM	Recycling Vending Machine
RVMs	Recycling Vending Machines
TAM	Technology Acceptance Model
PLS-SEM	Partial Least Squares Structural Equation Modeling
KMO	Kaiser–Meyer–Olkin
CR	Composite Reliability
α	Cronbach’s Alpha
AVE	Average Variance Extracted
VIF	Variance Inflation Factor
HTMT	Heterotrait–Monotrait Ratio
EVs	Electric Vehicles
MSW	Municipal Solid Waste
NBSC	National Bureau of Statistics of China
NFI	Normed Fit Index
SRMR	Standardized Root Mean Square Residual
